# Neurophthalmologic and Orthoptic Ambulatory Assessments Reveal Ocular and Visual Changes in Patients With Early Alzheimer and Parkinson's Disease

**DOI:** 10.3389/fneur.2020.577362

**Published:** 2020-11-03

**Authors:** Alessia Bargagli, Enrica Fontanelli, Dario Zanca, Ilaria Castelli, Francesca Rosini, Silvia Maddii, Ilaria Di Donato, Alessandra Carluccio, Carla Battisti, Gian M. Tosi, Maria T. Dotti, Alessandra Rufa

**Affiliations:** ^1^Neurosense-EVAlab DSMCN Università di Siena, Siena, Italy; ^2^UOC Oculistica Università di Siena, Siena, Italy; ^3^DIISM, University of Siena, Siena, Italy; ^4^UOC Neurologia e Malattie Neurometaboliche Università di Siena, Siena, Italy

**Keywords:** Alzheimer's disease, Parkinson's disease, retinal nerve fiber layer (RNFL), OCT, facial emotion recognition (FER), orthoptic rehabilitation

## Abstract

Patients with Alzheimer's disease (AD) and Parkinson's disease (PD) develop a progressive decline of visual function. This condition aggravates overall cognitive and motor abilities, is a risk factor for developing hallucinations, and can have a significant influence on general quality of life. Visual problems are common complaints of patients with PD and AD in the early stages of the disease, but they also occur during normal aging, making it difficult to differentiate between normal and pathological conditions. In this respect, their real incidence has remained largely underestimated, and no rehabilitative approaches have been standardized. With the aim to increase awareness for ocular and visual disorders, we collected the main neurophthalmologic and orthoptic parameters, including optical coherence tomography (OCT), in six patients with a diagnosis of PD, six patients with a diagnosis of early AD, and eight control subjects in an easily assessable outpatient setting. We also evaluated the patient's ability to recognize changes in facial expression. Our study demonstrates that visual problems, including blurred vision, diplopia, reading discomfort, photophobia, and glare, are commonly reported in patients with PD and AD. Moreover, abnormal eye alignment and vergence insufficiency were documented in all patients during examination. Despite the small size of the sample, we demonstrated greater ganglion cell and retinal nerve fibers layer (RNFL) damage and a defect of facial emotion recognition in AD/PD patients with respect to a comparable group of normal elderly persons, with peculiarities depending upon the disease. Ocular defects or visual discomfort could be correctly evaluated in these patients and possibly corrected by means of lens, orthoptic exercises, and visual rehabilitation. Such a practical approach may help to ameliorate motor autonomy, reading ability, and may also reduce the risk of falls, with a positive impact in daily living activities.

## Introduction

Neurodegenerative diseases are a heterogeneous group of clinical entities, often presenting with overlapping clinical pictures and characterized by progressive loss of neuronal populations in different brain regions including the visual system. The most relevant neurodegenerative entities in terms of prevalence and impact worldwide are Alzheimer's disease (AD) and Parkinson's disease (PD). Their socioeconomic relevance is currently high and is expected to grow up in the future. AD is an invariably fatal senile dementia with no cure and with a limited ability for early unequivocal diagnosis in primary care settings (1). AD is clinically represented by severe cognitive decline, sociobehavioral manifestations, and various visual dysfunctions (2, 3). Its neuropathology begins with the accumulation and propagation of misfolded amyloid β-protein (Aβ) assembly, followed by the hyperphosphorylation of (p)tau proteins, forming neurofibrillary tangles (NFTs) (4). These processes are associated with a cascade of secondary pathologic events, including inflammatory responses, vascular-associated abnormalities, oxidative stress, and mitochondrial dysregulation, leading to massive synaptic and neuronal loss (5–7). Importantly, growing evidence indicates that AD progressively spreads across the brain involving all visual system from the retina to primary and associative visual cortex (8, 9). PD is the second most common neurodegenerative disease, characterized by motor dysfunctions, such as tremor, rigidity, and bradykinesia, in addition to cognitive deficits, mood variability, and dementia (10, 11). Similar to AD patients, PD patients also complain of visual disturbances such as diplopia and hallucinations (12–14). The pathological hallmark of the diseases is the progressive accumulation of α-synuclein, a neurotransmitter involved in physical movement and reward-seeking behavior, and the depletion of cerebral dopamine. In recent years, various attempts of identifying surrogate clinical markers have been made with the aim of determining specific indicators of disease that may orientate early diagnosis and treatment or may have a prognostic value or improve the quality of life. The visual system from the peripheral sensory organs to the central integration system may be an early target of neurodegeneration in PD and dementia spectrum disorders. In both AD and PD, the progressive damage of visual function affects overall cognitive and motor abilities, is a risk factor for developing hallucinations, and can have a significant influence on general quality of life. Despite a broad variety of ocular disorders and visual problems being reported in patients with PD and AD, their systematic assessment has remained largely out of focus both in research and clinical practice.

With the aim to increase awareness for ocular and visual disorders, easily assessable in an outpatient setting, we collected the main neurophthalmologic and orthoptic parameters, including optical coherence tomography (OCT), in six patients with a diagnosis of PD, six patients with a diagnosis of AD, and eight control subjects. Moreover, in order to evaluate high-order visual function, we also assessed the ability to recognize changes in facial expressions. All patients were in the early stage of the disease and responded well to the treatment. Although ophthalmological examination in these patients frequently shows abnormalities, differences between PD and AD have never been evaluated previously. Our study further supports the idea that visual disturbances are common in PD and AD patients. Moreover, we found a significant damage of ganglion cells (GCs), retinal nerve fibers layer (RNFL), and a defect in facial emotion recognition, with peculiarities depending upon the disease. Ocular misalignment and vergence insufficiency or visual discomfort could be correctly evaluated in these patients and possibly corrected by means of lens, orthoptic exercises, and visual rehabilitation. This practical approach improves motor autonomy, that is, the ability of the subject to move autonomously in the surrounding environment to perform common daily activities.

## Materials and Methods

### Subjects

We evaluated six AD patients (average age of 69, from 53 to 78 years) and six PD patients (average age of 74, from 66 to79 years), referring to our outpatient service of neurophthalmology and orthoptic.

The diagnosis of AD was confirmed by F-flutemetamol-labeled amyloid positron emission tomography (PET). Mean mini-mental state examination (MMSE) was 23.2 ± 3.3 (range 17–28). All AD patients were self-sufficient. [Activities of daily living (ADL) were normal for all AD patients and mean IADL score was 6.8], and all of them were taking pharmacological therapy (cholinesterase inhibitors).

PD patients were defined as idiopathic Parkinson and staged using a Unified Parkinson's disease rating scale, UPDRS III (mean 15.3 ± 4.7 range 21–10). The diagnosis was confirmed by dopaminergic single-photon emission computed tomography and magnetic resonance imaging (MRI), which excluded vascular PD. All PD patients who were taking Levodopa and dopamine agonists had only bradykinesia (no tremor) and normal MMSE.

The control group included eight age- and sex-matched healthy subjects, free from treatments affecting ocular or neurological functions and without a past history of ocular or neurological diseases. Controls did not have neurological disturbances or cognitive impairment at time of the evaluation. Informed written consent was obtained from all study participants. The study was performed according with the criteria of the Declaration of Helsinki, and it was approved by the local Ethical Committee Azienda Ospedaliera Universitaria Senese, EVAlab protocol CEL no. 48/2018.

### Neurophthalmologic Examination

Each subject undergone to a complete *neurophthalmologic* ambulatorial examination including best corrected visual acuity (Snellen's chart), color sensitivity (Ishihara testing), Amsler chart, confrontation visual field, pupil's reactivity, slit lamp, tonometry (Tonopen), ocular motility, and ophthalmoscopy.

### Orthoptic Examination

Orthoptic examination included non-dissociating and dissociating tests: Hirshberg corneal reflexes, assessing alignment for near (35 cm) and distance (5 m), and stereopsis tested by Lang I stereotest with near glasses. Binocularity was evaluated with Worth 4-dot test at near and distance. Cross-cover test with prisms were performed at both distances to measure the magnitude of deviation. Near point of convergence (NPC) was measured, gradually approaching a light target, from a distance of 50–60 cm, starting from the bottom, with an inclination of 20° with respect to primary visual direction. A convergence insufficiency (CI) was diagnosed with NPC ≥ 10 cm, and NPC was considered remote when it was 25 cm or more; duction/version movements were examined in all cardinal gaze positions. Conjugate eye movements, including saccades, pursuit, VOR, vergence, and fixation, were evaluated clinically.

### Ocular Coherence Tomography (OCT)

The exam was completed by performing an OCT (ZEISS cirrus HD 5000). Three scan programs were selected: macular thickness (macular cube 512 × 128), RNFL and OHN analysis (optic disc cube 200 × 200), GC analysis (macular cube 512 × 128). The central macular thickness, average RNFL, average RNFL of the temporal, nasal, superior, and inferior nasal sectors, the average value of GCs, and average values of GCs in nasal, superior, inferior, and temporal sectors were considered. All data were acquired at the Ophthalmology Department of the University of Siena, by an experienced ophthalmologist blind to the clinical status of the subjects. Patients' values were compared with those of a group eight healthy age- and sex-matched subjects. Exclusion criteria for controls were systemic metabolic diseases, cerebrovascular risk factors, and history of neurological, cardiac, and ophthalmological disorders including glaucoma (intraocular pressure <21 mm Hg) and myopia <3 diopters.

### Facial Emotion Recognition

We evaluated the subject's ability to recognize a change in facial emotion expression using Facial Emotion Recognition (FER) software developed at the Eye Tracking and Visual Application Lab (EVAlab, http://evalab-eyetech.com/), University of Siena. The FER software manages a dataset of facial morphing on people who change their expressions from neutral to six different expressions. Morphing was obtained using the Ekman's database of human faces, balanced in terms of age and sex. The study consisted in a prime test with six trials of training, including six basic emoticons presented to the subject. Each task consisted of 10 trials in which the subject has to recognize an emotion while a morphing slowly changes its expression at a fixed velocity (within 4 s) from the neutral expression to one of the six basic Ekman's expressions (happiness, sadness, anger, fear, surprise, and disgust). The patient was instructed to press the button when he can recognize the forthcoming expression, then he had to confirm his choice by selecting the corresponding emoticon. The software keeps track of all responses, correct or incorrect, as well as the reaction times.

### Statistical Analysis

The normality of distribution of the investigated parameters has been assessed using the Kolmogorov Smirnov test. All parameters in our study are distributed normally. Data is expressed as mean ± standard deviation. One-way analysis of variance is performed to identify significant differences between groups. A threshold of 0.05 is considered for significance. When differences are found, the results of pairwise *post-hoc t*-test are provided. Pearson's correlation (*p*-value) is used to analyze the difference between the sample means. The values *p* < 0.05 were considered statistically significant.

## Results

### Neurophthalmological and Orthoptic Results

At the time of neurophthalmologic and orthoptic evaluation, one AD patient reported a past history of blepharospasm treated with botulin toxin, while all patients reported blurred vision, reading difficulties, and photophobia. Visual troubles persisted after cataract surgery in two patients.

Best corrected visual acuity was normal in all patients (20/20 to 20/25). Color vision and confrontational visual field was normal and did not differ between groups. Pupils were reactive to the light and near in all patients, tonometry, and anterior segment was in normal range for all groups. All patients reported lacrimation, photophobia, and glare from mild to severe. No ocular deviation emerged for any group, during the corneal light reflex examination. Stereopsis was also present, and a single binocular vision was reported in Worth light test.

The cover test with prisms demonstrated a slight eso/exo-phoria with slow recovery and possibility of dissociation in phoria/tropia in both patient's groups. Five up to six PD patients presented exophoria for near (mean 12.4^∧^ range 6–18^∧^) and distance (mean 2^∧^ range 2–4^∧^); three AD patients showed esophoria for near (mean 5^∧^ range 4–6^∧^) and far (mean 6^∧^ range 4–8^∧^); the other three displayed exophoria for near (mean 8^∧^ range 6–12^∧^) and only one of these for far too (6^∧^) (see Table 1).

**Table 1:** Orthoptic evaluation: Corneal reflexes for near and far, cross-cover test quantified by prismatic diopters, versions' test to evaluate ocular motility and near point of convergence.

**Disease**	**Corneal light reflex**	**Cross cover test**	**Versions**	**NPC**
PD	Normal	Exophoria 12^∧^ near	Inside limits	Remote
PD	Normal	Exophoria 6^∧^ near and 2^∧^ far	Inside limits	15 cm
PD	Normal	Exophoria 12^∧^ near 4^∧^ far	Inside limits	Remote
PD	Slight exoforia for near	Exophoria 18^∧^ near 4^∧^ far	Hypofunction medial recti	Remote
PD	Normal	Exophoria 14^∧^ near	Hypofunction medial recti	Remote
PD	Normal	Normal	Inside limits	Normal
AD	Normal	Exophoria 6^∧^ near	Hypofunction medial recti	13 cm
AD	Normal	Esophoria 4^∧^ near 8^∧^ far	Hypofunctions lateral recti	Normal
AD	Normal	Exophoria 6^∧^ near	Inside limits	14 cm
AD	Normal	Normal	Hypofunction inferior oblique	Normal
AD	Normal	Exophoria 12^∧^ near exot 6^∧^ far	Hypofunction medial recti	Remote
AD	Normal	Esophoria 6^∧^ near 4^∧^ far	Inside limits	Normal

A slight hypofunction of the medial recti was found in all PD patients, as well as in two AD patients. Almost all patients with exodeviation had a greater exophoria for near and CI, with an NPC break of >10 cm. Patients with CI complained of intermittent diplopia, general discomfort, and blurred vision for near, especially when reading. Clinical examination of the conjugate eye movements (saccades, pursuit, and VOR) did not show any evident limitation.

### OCT Macular, Optic Disc, Ganglion Cells Analysis

**Figure 1:**
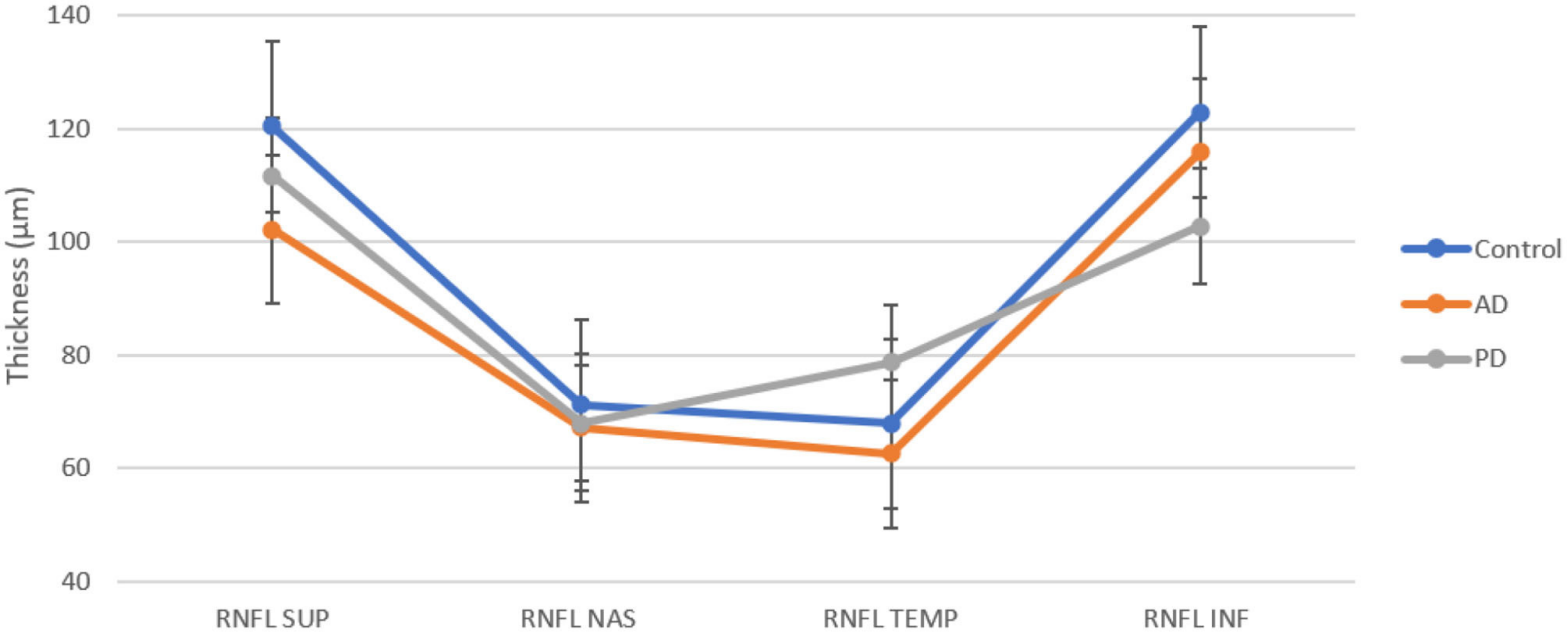
RNFL thickness in superior, nasal, temporal, and inferior sectors for each group.

The average value of RNFL was 86.91 ± 2.25 μm in AD, 89.67± 8.78 μm in PD, and 95.63 ± 8.65 μm in controls (see Table 2). A one-way analysis of variance showed significant RNFL thickness differences in the superior sector, *F*(2, 17) = 3.67, *p* < 0.05. *Post-hoc* analyses using a *t*-test showed a significant thinning of the superior quadrants in AD patients with respect to controls (*p* = 0.0059). We did not observe any significant differences between AD and PD (see Figure 1).

**Table 2:** RNFL values for each quadrant (superior, nasal, inferior, temporal) recorded at OCT. Average and standard deviation are reported for each group.

	**RNFL SUP**	**RNFL NAS**	**RNFL TEMP**	**RNFL INF**
AD	**102.25** **±** **11.5**	67.25 ± 5.55	62.67 ± 8.69	115.83 ± 12.58
PD	111.75 ± 14.57	68 ± 7.78	78.75 ± 30.14	**102.83** **±** **19.47**
Control	120.5 ± 11.5	71.25 ± 12.8	67.89 ± 8.56	122.94 ± 19.81

*In bold, values that are significantly different from those of the control population (p < 0.05)*.

*All values are measured in μm*.

The average value of the macular cube was 262 ± 12.58 μm in AD, 279.5 ± 20.12 μm in PD patients, and 289.31 ± 12.40 μm in controls. A one-way analysis of variance did not show a significant difference of the macular cube size among groups, *F*(2, 17) = 2.22, *p* = 0.14.

**Figure 2:**
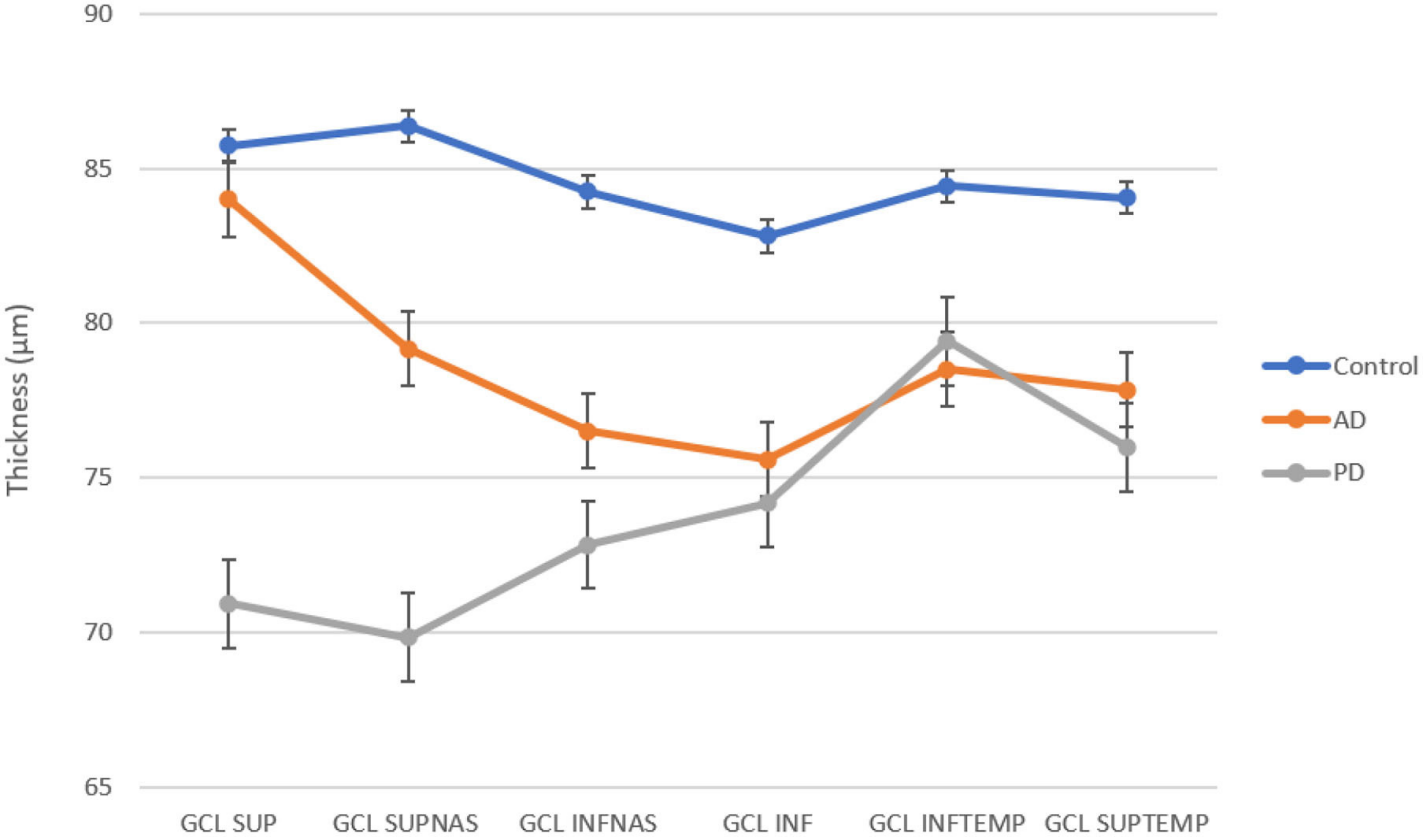
GCL thickness in superior, superiornasal, inferiornasal, inferior, inferiortemporal, and superiortemporal sectors for each group. AD and PD patients show a reduction in all sectors with respect to control.

The mean value of ganglion cell layer (GCL) thickness was 78.25 ± 8.44 μm in AD, 73.75 ± 7.94 μm in PD, and 84.56 ± 4.81 μm in the control group (see Table 3). A one-way analysis of variance showed significant differences in the superior sector, *F*(2, 17) = 6.42, *p* = 0.008, nasal superior sector, *F*(2, 17) = 6.63, *p* = 0.007, and nasal inferior sector, *F*(2, 17) = 3.98, *p* = 0.038. *Post-hoc* analysis evidenced a significant reduction of the GCL thickness in nasal superior sector between AD and Controls (AD 79.17 ± 8.09 μm; Controls 86.36 ± 4.96 μm; *p* = 0.0455); and between PD and Controls (PD 69.83 ± 11.86 μm; Controls 86.36 ± 4.96 μm; *p* = 0.0085); in nasal inferior sector between PD and Controls (PD 72.83 ± 9.53 μm; Controls 84.25 ± 4.59 μm; *p* = 0.016). The GCL of the nasal superior sector was significantly reduced in PD patients with respect to AD (*p* = 0.0201) (see Figure 2).

**Table 3:** GCL average and standard deviation for each sector (superior, superiornasal, inferiornasal, inferior, superiortemporal).

	**GCL SUP**	**GCL SUP NAS**	**GCL INF NAS**	**GCL INF**	**GCL INF TEMP**	**GCL SUP TEMP**	**Macular cube**
AD	84 ± 8.44	**79.17** **±** **8.08**	76.5 ± 9.23	75.58 ± 8.94	78.5 ± 10.32	77.83 ± 9	**272.33** **±** **12.58**
PD	**70.92** **±** **10.53**	**69.83** **±** **11.86**	**72.83** **±** **9.53**	**74.17** **±** **7.57**	79.42 ± 6.88	**76** **±** **4.71**	279.5 ± 20.12
Control	85.75 ± 5.42	86.38 ± 4.96	84.25 ± 4.59	82.81 ± 5.28	84.44 ± 5.27	84.06 ± 5.21	289.31 ± 12.40

*In the last column, the average and standard deviation of macular cube. In bold, the values are significantly different from those of the control population (p < 0.05)*.

*All values are measured in μm*.

### FER Results

Both AD and PD patients performed significantly worse than the control group in the task of human facial expressions recognition. The total number of errors per test was 15 ± 9.19 in AD; 16 ± 9.44 in PD; and 2.9 ± 2.70 in Control. A one-way analysis of variance showed significant differences in the performance of the different groups in terms of total number of errors, *F*(2, 17) = 7.11, *p* = 0.006. *Post-hoc* analysis using *t*-tests showed significant differences between AD and Control (*p* = 0.01); and between PD and Control (*p* = 0.009).

Although they fall slightly outside the chosen range of significance, some interesting differences for specific subsets of facial expressions have been observed: fear, *F*(2, 17) = 3.37, *p* = 0.06; surprise, *F*(2, 17) = 2.89, *p* = 0.08; and sadness, *F*(2, 17) = 3.25, *p* = 0.06. *Post-hoc* analysis, evidenced that PD struggle in recognizing fear expressions with respect to Control (*p* = 0.037). Differently, sadness was the least recognized emotion in AD patients (*p* = 0.0362), followed by fear, surprise, anger, and disgust. Surprise was consistently confused with anger by PD patients (*p* = 0.038) and with fear by AD patients (*p* = 0.027).

## Discussion

Almost 80% of visual impairments worldwide are treatable or preventable; thus, timely recognition is pivotal in the general population (15). An intact vision is crucial in AD to preserve as long as possible visual-dependent cognitive functions, and in PD patients for compensating, through visual guidance, the loss of motor automaticity and postural instability. In this study, we confirm that ocular and visual problems commonly occur in patients with AD and PD, compared to healthy subjects of the same age who come to a neurophthalmologic outpatient service. Similar pathological involvement of the visual system is present in both diseases but also in aging. Nevertheless, in our study, differences emerged between the groups. Large population studies have demonstrated a greater prevalence of age-related visual problems among elderly people with mild cognitive impairment/dementia and PD (16, 17) compared to normal individuals (18, 19).

Ocular and visual changes, in terms of incidence, prognosis, and clinical relevance, have been extensively investigated in each disease; nevertheless, there are very few reports comparing them in a practical outpatient setting. The results of our study demonstrate that visual acuity, color vision, and confrontation visual field were in the normal range in all patients; rather, they complained more subtle difficulties, regarding the quality of their vision such as inconstant double or blurred vision and visual discomfort when reading, asthenopia, photophobia, and glare, which were not reported by our control group. We also found a higher prevalence of latent eye deviations (heterophorias, exo/eso-phorias) with larger angle of deviation in PD than AD. All PD patients showed exophorias greater in near viewing, which was associated to CI, remote NPC, and inconstant diplopia. Unlike PD, AD patients had small exo- or eso-phorias and less affected convergence amplitude. It has been reported that more than 20% of PD patients experience intermittent diplopia, which is mostly associated to oculomotor abnormalities and less frequently, due to impaired vision or visual hallucinations (13). The most prevalent oculomotor abnormalities causing misalignment in PD are those related to fusional disorders, including convergence insufficiency, decompensating exophoria, and reduced fusional range. This general impairment of vergence eye movements results from basal ganglia dysfunctions and improve with dopaminergic replacement therapy (20). On the other hand, daily fluctuations in dopamine levels may, in turn, produce inconstant convergence insufficiency. The prevention of dopamine fluctuations associated to orthoptic exercise and prisms may alleviate visual problems in these patients (20, 21). In our experience, photophobia, glare, and dry eye may be alleviated using lenses with specific filters, artificial drops, and cataracts surgery.

Contrary to PD, reading difficulties reported by AD patients are less likely related to convergence insufficiency and fusional disorder but may indicate an early cortical disturbance of visual perception or sensory motor integration. Early pathological changes in visual integration areas may produce subtle deficit of visual spatial attention or ambiguity in distinguishing letters, object shapes, or orientation. Overall, these symptoms are distinctive features of the posterior variant of dementia, but, even if less prominent, may also be present in the more common variants of AD (17, 22). Rehabilitation of visual attention and other cortical visual functions using eye tracking and orthoptic exercises have been suggested to reduce the impact of these symptoms on patient's autonomy (17).

In this study, we also demonstrate a reduction of the RNFL and GCL in both patient's groups with respect to normal controls with further differences distinguishing the two diseases. In particular, we found a global decrease of the RNFL thickness in both patients compared to controls. Analogously to what was reported in the literature, the RNFL thinning is typically localized in the superior and inferior quadrants without significant differences among groups (see Figure 1) (23, 24).

A more sensitive parameter in differentiating the three groups was the GCL thickness. According to previous OCT studies (25–27), we demonstrated a GC loss in the superior quadrants of AD patients with respect to both PD and controls; however, due to the small sample, the difference did not reach the level of significance. Figure 2 shows a different trend of the curves representing GCL thickness across retinal quadrants. Several post-mortem studies and animal models have revealed the AD-specific hallmarks in the retina. Aβ plaques and hyperphosphorylated (p)tau were particularly abundant in the inner retinal layers, especially in the GCL (28) and co-localized around and within degenerating retinal GCs (29). Furthermore, multiple recent studies into region-specific retinal degeneration in AD patients showed that the most significant damage of the inner retinal layer and RNFL are localized in far and midperiphery of the superior quadrant (30, 31). Analogously, studies examining the retina of PD patients by OCT have revealed a significant thinning of macular volume (32–34) and a decrease of GCL and RNFL thickness mostly in inferior and temporal quadrants (23, 24, 35, 36) compared to healthy subjects.

In both diseases, OCT parameters have been correlated with clinical scores of disease progression supporting the important role of OCT in monitoring both PD and AD evolution (37).

**Figure 3:**
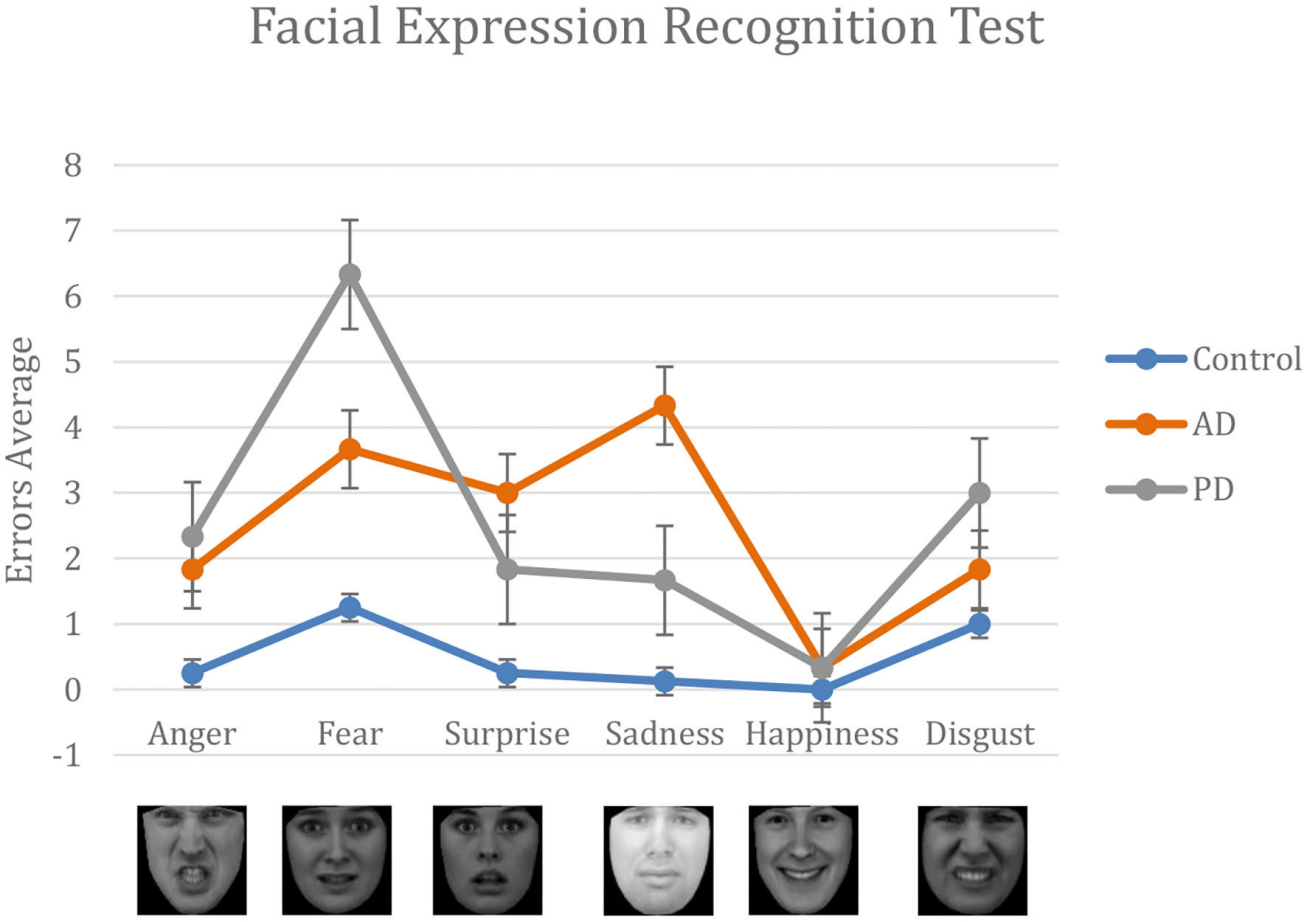
Results from the FER test. For each emotion, we compare the number of errors made by AD, PD, and the control group. Performance of AD and PD are systematically worse than the control group. Happiness is the easiest emotion recognized by all group.

Finally, all patients demonstrated a difficulty in the recognition of facial expression changes, particularly for negative emotions. In particular, AD patients showed a significant higher number of errors in identifying the correct emotion and a longer latency than PD, and both did worse than the controls. Recognition of emotional expressions is considered an important prerequisite for interpersonal functioning and quality of life. Emotion perception has been shown to rely on a ventral affective system, including the hippocampus, amygdala, insula, ventral striatum, and ventral regions of the anterior cingulate gyrus and prefrontal cortex (38). Regions in the ventral system, including the amygdala, are susceptible to atrophy in PD and AD at any stage. Both groups experienced difficulty with negative emotions recognition (see Figure 3) compared with controls. In a quantitative MRI study, a direct correlation has been found between temporal volume and low score in negative facial expression recognition. However, while previous reports agree with the observation that negative emotions are poorly recognized in AD and PD, conflicting results have been obtained when searching for a specific emotion (39, 40). Most studies on facial expression recognition have been made using a static image presentation, which is a less naturalistic and less-sensitive approach. In this study, we proposed an emotion-intensity-based approach using an interactive software that is more sensitive to identify subtle impairment in detecting specific facial expression changes. Moreover, most studies suggest that deficits in emotion perception occur in the context of unimpaired face perception (41); however, we found a significant longer latency in the identification of expression changes, suggesting almost in part, a slow facial perception or processing load in both pathological conditions, particularly AD.

A limitation of the study is the small sample; however, even if further studies will be necessary to confirm the differences between groups, it is evident that visual deficits are common, occur early, and impact the quality of life in both diseases. Early identification of visual changes is useful for developing interventions that are specifically targeted at these core visual problems in AD and PD patients. Among intervention, orthoptic rehabilitation may help to minimize ocular symptoms, contributing to improve every activities of day living. A home and outpatient exercise program, based on NPC and vergences training, pencil push-ups, prisms, and 3D stereograms exercises, is an effective treatment in convergence insufficiency. Prismatic lenses can be a satisfactory solution in case of subjective diplopia. In conclusion, clinicians should be encouraged to consider orthoptic rehabilitation, a therapy with minimal risk, in patients with neurodegenerative disease whose quality of life is affected by binocular dysfunction.

## Data Availability Statement

The raw data supporting the conclusions of this article will be made available by the authors, without undue reservation.

## Ethics Statement

The studies involving human participants were reviewed and approved by Ethical Committee Azienda Ospedaliera Universitaria Senese, EVAlab protocol CEL no. 48/2010. The patients/participants provided their written informed consent to participate in this study.

## Author's Note

We would like to thank IC who, in life, has contributed with the realization of the morphing videos with human facial expressions, leveraging an implementation of Active Appearance Models (AAMs, originally by Stefano Melacci). She was among the first to understand their possible applications in medical and rehabilitative fields.

## Author Contributions

AR, AB, and EF conceived of the study. IC lead the software project of the morphing faces. AB and EF collected the data under the supervision of GT, MD, AC, ID, CB, and AR. AB, EF, and DZ analyzed the data. The paper was drafted by AB, EF, DZ, SM, and AR. All authors provided detailed comments.

## Conflict of Interest

The authors declare that the research was conducted in the absence of any commercial or financial relationships that could be construed as a potential conflict of interest.

## Abbreviations

AD: Alzheimer's disease
CI: convergence insufficiency
FER: facial emotion recognition
GCL: ganglion cells layer
MMSE: mini mental state examination
MRI: magnetic resonance imaging
NPC: near point convergence
OCT: optical coherence tomography
PET: positron emission tomography
PD: Parkinson disease
RNFL: retinal nerve fibers layer
UPDRS: Unified Parkinson's disease rating scale.
